# MiR-542-5p is a negative prognostic factor and promotes osteosarcoma tumorigenesis by targeting HUWE1

**DOI:** 10.18632/oncotarget.6199

**Published:** 2015-10-20

**Authors:** Dong-dong Cheng, Tao Yu, Tu Hu, Ming Yao, Cun-yi Fan, Qing-cheng Yang

**Affiliations:** ^1^ Department of Orthopedics, Shanghai Jiao Tong University Affiliated Sixth People's Hospital, Shanghai, China; ^2^ State Key Laboratory of Oncogenes and Related Genes, Shanghai Cancer Institute, Renji Hospital, Shanghai Jiao Tong University School of Medicine, Shanghai, China

**Keywords:** miR-542-5p, proliferation, proteomics, HUWE1, osteosarcoma

## Abstract

Recent evidence has demonstrated that microRNAs (miRNAs) are involved in the proliferation and metastasis of osteosarcoma. Using miRNA microarray and functional screening methods to compare miRNA expression profiles in osteosarcoma cell lines treated with Trichostatin A (TSA), overexpression of miR-542-5p was determined to be involved in the proliferation of osteosarcoma. We used isobaric tags for relative and absolute quantitation (iTRAQ) and nanoscale liquid chromatography-mass spectrometry (NanoLC−MS/MS) to identify differentially expressed proteins in MNNG/HOS and U2OS osteosarcoma cell lines transfected with miR-542-5p; in both cell lines, seven proteins were downregulated, and nine were upregulated. HUWE1 was found to be a direct target of miR-542-5p in both osteosarcoma cell lines, and was negatively correlated with miR-542-5p levels in human osteosarcoma tissues. Moreover, the expression of miR-542-5p was upregulated in human osteosarcoma tissue compared with non-tumor adjacent tissue. Kaplan-Meier analysis revealed that overexpression of miR-542-5p predicted poor prognosis for osteosarcoma patients. Taken together, our results indicated that miR-542-5p plays a critical role in the proliferation of osteosarcoma and targets HUWE1.

## INTRODUCTION

Osteosarcoma is the most common type of malignant bone tumor, and it frequently originates in the metaphysis of the long bones [1]. In the past, the most common treatment for osteosarcoma was amputation. While the 5-year survival rate for patients with osteosarcoma has significantly improved, many patients are insensitive to available chemotherapeutics and have poor prognoses [2].

MiRNAs are a class of small regulatory RNA molecules, 21-23 nucleotides in length, that negatively regulate the expression of their target genes by binding to the 3′-untranslated regions (UTR) of the corresponding mRNAs [3]. Previous studies have reported that miRNAs can act as oncogenes or tumor suppressors during the development and metastasis of osteosarcoma [4, 5]. MiR-93 reportedly promotes the metastasis of osteosarcoma by targeting E2F1 [6]. Zhang *et al*. [7] found that miR-143 expression was low in osteosarcoma specimens and in cultured osteosarcoma cell lines, and that overexpression of miR-143 reduced cell viability, induced apoptosis, and inhibited tumor growth via Bcl-2 *in vitro*.

The histone deacetylase inhibitor trichostatin A (TSA) reportedly exerts an anti-tumor effect by increasing levels of histone acetylation [8]. In that regard, TSA alters the miRNA expression profiles of breast cancer cells [9], and inhibits cell proliferation and metastasis in osteosarcoma [10]. The purpose of the present study was to determine the effects of TSA on the miRNA expression profile in osteosarcoma and to identify miRNAs that enhance the development of this cancer. Our findings suggest miR-542-5p enhances the growth of osteosarcoma cells by targeting HUWE1 and may provide a potentially useful therapeutic target for treatment of osteosarcoma.

## RESULTS

### The influence of TSA on miRNA expression in osteosarcoma cells

**Figure 1:**
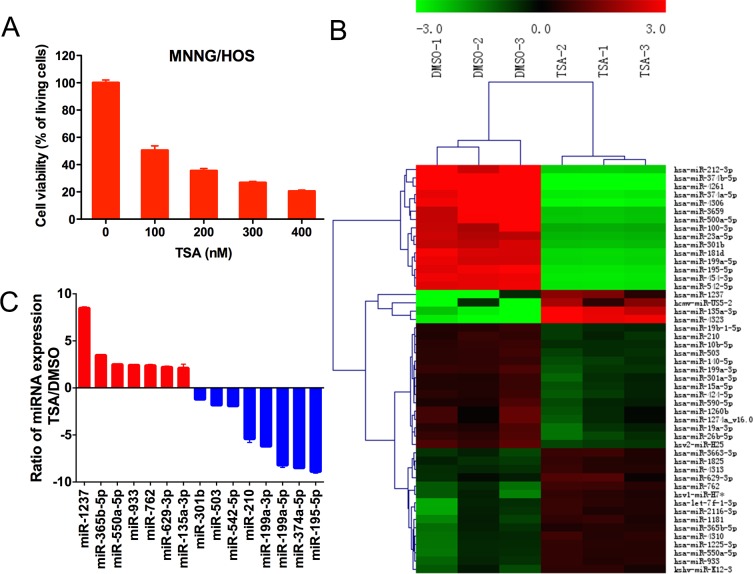
The influence of TSA on miRNA expression in MNNG/HOS cells **A.** Cell viability was quantified with CCK-8. The TSA concentration selected for the rest of the study was 110 nmol/L for MNNG/HOS cells, which were treated for 48 hours. **B.** Heat map of miRNAs that are differentially expressed between the TSA-treated group and the DMSO-treated group. Three biological replicates were analyzed. **C.** Quantitative real-time RT-PCR validation of seven upregulated and eight downregulated miRNAs.

Microarray analysis was used to characterize the TSA-induced miRNAs. Cell viability was quantified with CCK-8. The half maximal inhibitory concentration (IC50) of TSA for MNNG/HOS cells was 110 nmol/L (Figure 1A), and this concentration was used for the remainder of the study. Microarray data revealed that after TSA treatment, 19 miRNAs, including miR-4323 and miR-135a-3p, were upregulated 2-fold relative to the control group; 30 miRNAs, including miR-4261, miR374b-5p and miR-4306, were downregulated 2-fold (Figure 1B). We performed GO analysis and KEGG pathway analysis on the differentially expressed miRNAs, and the results are shown in Supplementary Figure 1. Ultimately, fifteen potential miRNAs associated with the proliferation of osteosarcoma were selected for microarray validation by quantitative real-time RT-PCR analysis, including miR-1237, miR-550a-5p, miR-365b-5p, miR-135a-3p, miR-933, miR-762, miR-629-3p, miR-542-5p, miR-503, miR-301b, miR-210, miR-374a-5p, miR-199a-5p, miR-199a-3p and miR-195-5p. Changes observed via qRT-PCR were consistent with the microarray analysis (Figure 1C).

### Functional screening by *in vitro* transfection of miRNA mimics and inhibitors

**Figure 2:**
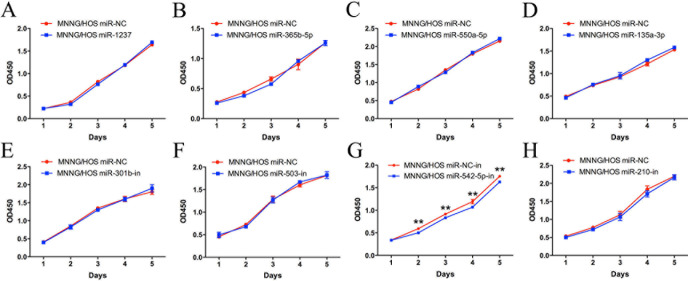
Functional screening of eight candidate miRNAs in the MNNG/HOS cell line A CCK-8 assay was used to detect the proliferation of MNNG/HOS cells after transfection with miRNA mimics or inhibitors. Error bars represent the SEM, ** *P* < 0.01.

MiRNA mimics for miR-1237, miR-365b-5p, miR-550a-5p and miR-135a-3p and miRNA inhibitors for miR-301b, miR-503, miR-542-5p and miR-210 were chosen for further functional investigation. MNNG/HOS cells were transiently transfected with miRNA mimics or inhibitors and a CCK-8 assay was used to detect changes in proliferation. The results showed that inhibition of miR-542-5p could restrain the proliferation of tumor cells, whereas the other miRNAs had no discernible effect on the proliferation of MNNG/HOS cells (Figure 2).

### MiR-542-5p promotes the proliferation of osteosarcoma cells *in vitro*

**Figure 3:**
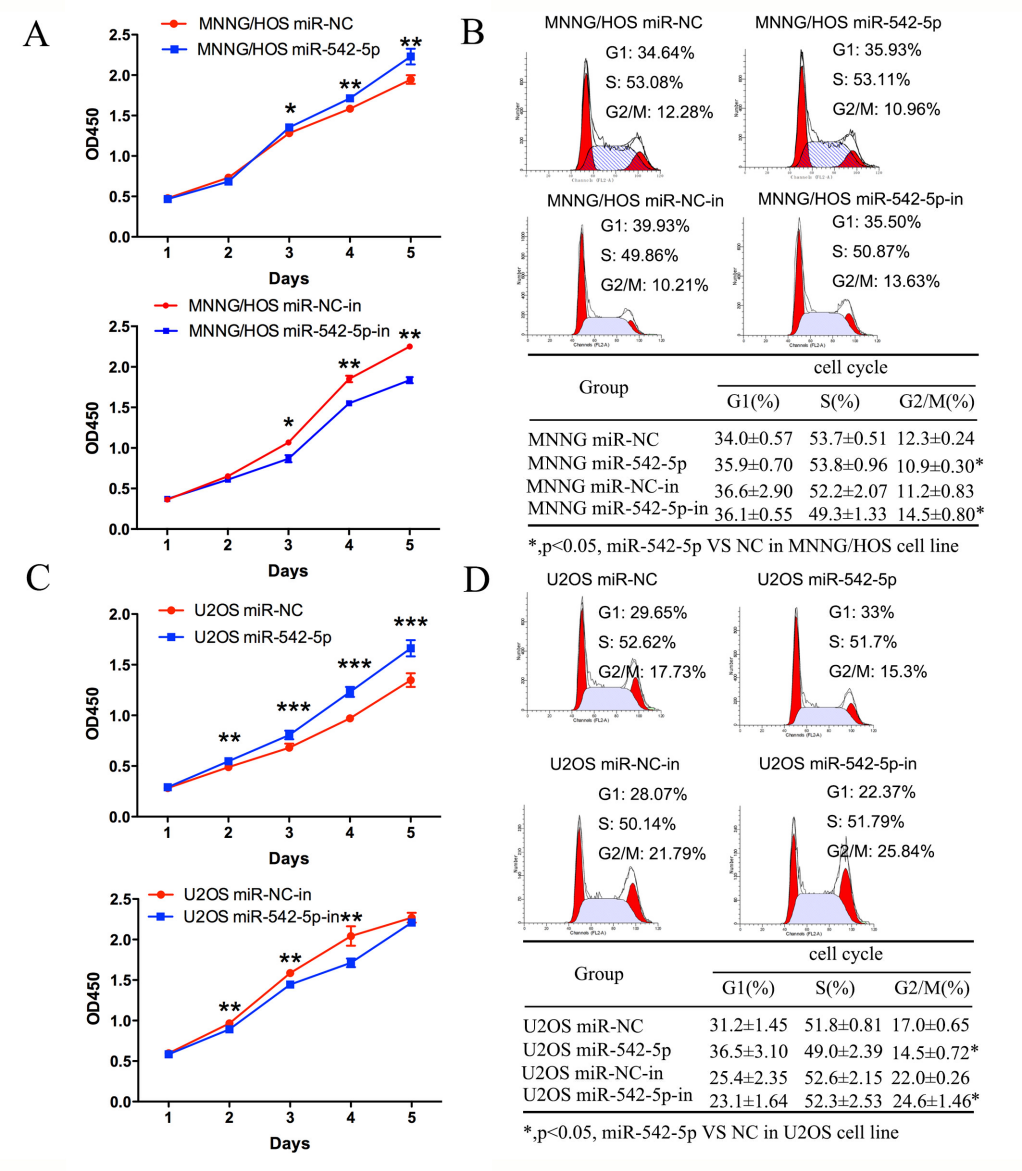
Effects of miR-542-5p on the proliferation and cell cycle progression of osteosarcoma cells **A.** Diagrams showing the results of a CCK-8 assay in MNNG/HOS cells transfected with miR-542-5p/miR-NC or miR-542-5p-in/miR-NC. **B.** Representative images and a table depicting the results of cell cycle assays in MNNG/HOS cells transfected with miR-542-5p/miR-NC or miR-542-5p-in/miR-NC. **C.** Diagrams showing CCK-8 assay results in U2OS cells transfected with miR-542-5p/miR-NC or miR-542-5p-in/miR-NC. **D.** Representative images and a table depicting the results of cell cycle assays in U2OS cells transfected with miR-542-5p/miR-NC or miR-542-5p-in/miR-NC. All results are shown as the mean ± standard error of the mean (SEM). *P < 0.05; **P < 0.01. OD, optical density.

**Figure 4:**
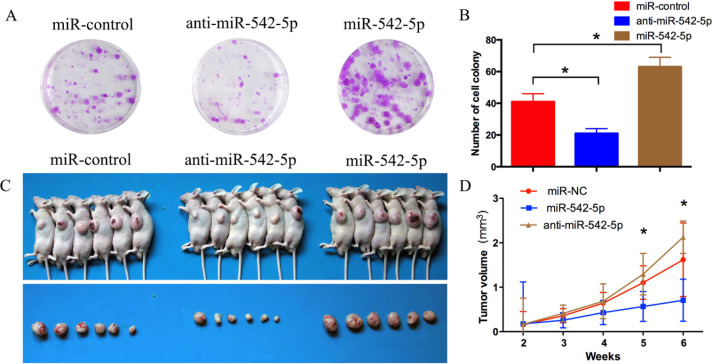
MiR-542-5p promotes the growth of osteosarcoma tumors *in vivo* **A. B.** Colony formation assay of MNNG/HOS cells stably expressing the miR-542-5p mimic, miR-542-5p inhibitor or miR-control. After 2 weeks, cells in each well were fixed and counted. **C.** Photographs of tumors. **D.** Growth curve drawn by measuring tumor volumes on the indicated days. Error bars represent the SEM, *P < 0.05.

MNNG/HOS and U2OS cells were transiently transfected with a miR-542-5p mimic or inhibitor to increase or decrease expression of the miRNA. The relative expression of miR-542-5p was shown in Supplementary Figure 2. A CCK-8 assay was used to measure cell proliferation. The results showed that upregulation of miR-542-5p promoted osteosarcoma cell proliferation; in contrast, knockdown of miR-542-5p had the opposite effect (Figure 3A, 3C). Cell cycle changes were analyzed by flow cytometry after transfection with the miR-542-5p mimic or inhibitor. Results of cell cycle analysis revealed that overexpression of miR-542-5p resulted in a reduced G_2_/M population in MNNG/HOS and U2OS cells, whereas inhibition of miR-542-5p inhibited cell cycle progression, with increased numbers of cells in G_2_/M phase (Figure 3B, 3D). Thus, miR-542-5p may enhance cell proliferation by promoting the G_2_/M phase transition. MNNG/HOS cell lines stably expressing the miR-542-5p mimic, the miR-542-5p inhibitor or the miR-control were established. Colony formation assays showed that overexpression of miR-542-5p promoted colony formation and inhibition hindered colony formation (Figure 4A, 4B). However, miR-542-5p did not influence the migration or invasion of osteosarcoma cells *in vitro*, as shown in Supplementary Figure 3.

### MiR-542-5p promotes tumor formation *in vivo*

MNNG/HOS cells stably expressing the miR-542-5p mimic, the miR-542-5p inhibitor or the miR-control were subcutaneously injected into the left scapulas of nude mice, and the animals were closely monitored for tumor growth for 6 weeks. The results demonstrated that miR-542-5p-overexpressing tumors were significantly larger in size and volume compared with control tumors, whereas miR-542-5p-underexpressing tumors were smaller in size and volume compared with control tumors (Figure 4C, 4D).

### HUWE1 is the direct downstream target of miR-542-5p

**Figure 5:**
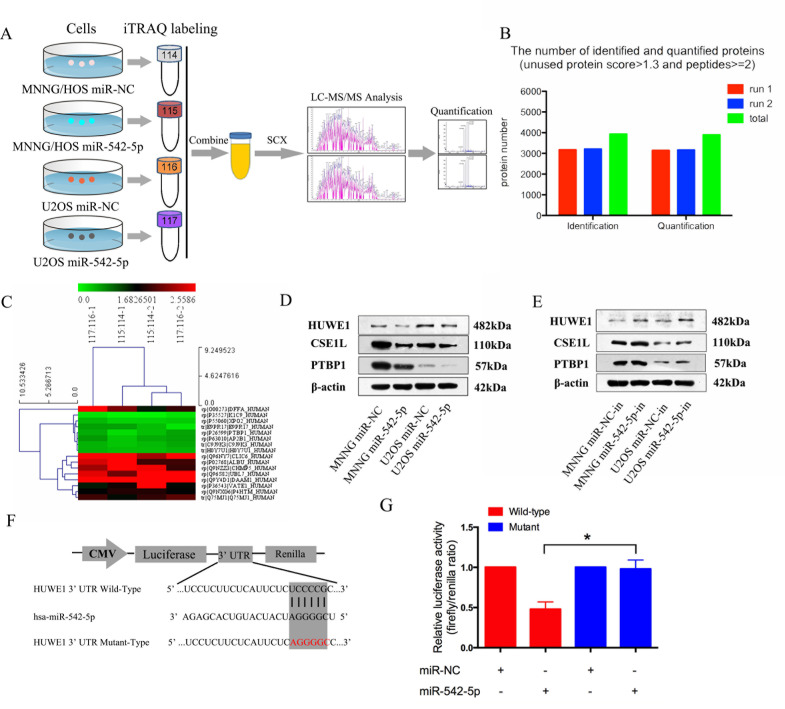
HUWE1 is the direct downstream target of miR-542-5p **A.** Flow chart of proteomic analysis. **B.** Total identified and quantified proteins. All proteins had an unused protein score >1.3 and peptides ≥2. **C.** Heat map of upregulated and downregulated proteins in both MNNG/HOS and U2OS cells after transfection with miR-542-5p. **D. E.** Representative western blots displaying the levels of HUWE1, CSE1L and PTBP1 proteins after transfection with miR-542-5p mimics or inhibitors. β-actin was used as an internal control. **F.** The sequences of the putative miR-542-5p binding sites in wild type (emphasized with shadow) and mutant (red) HUWE1-3′UTR. **G.** The relative luciferase activity of luciferase reports with wild type or mutant HUWE1-3′UTR were determined in HEK 293T cells, which were co-transfected with the miR-542-5p mimic or miR-NC. Renilla luciferase activity served as an internal control. Statistical significance was observed between the wild type and the mutant groups transfected with miR-542-5p. The data are representative of three independent experiments. Error bars represent SEM. * *P* < 0.05 by Student's *t* test.

iTRAQ, combined with NanoLC−MS/MS analysis, was used to identify proteins that were differentially expressed in MNNG/HOS and U2OS cells after transfection with the miR-542-5p mimic. Using ProteinPilot 4.1, we identified a total of 4078 and 3989 proteins and 36543 and 35941 peptides (global FDR < 1%) in the first and second runs, respectively. After filtering for an unused protein score > 1.3 and the number of peptides ≥ 2, 3178/3210 proteins were identified and 3147/3167 proteins were quantified. In total, we identified 3938 proteins and quantified 3898 proteins in the two replicates (Figure 5B). We identified nine upregulated (both of the iTRAQ ratios 115:114 > 1.5 and 117:116 > 1.5) and seven downregulated proteins (both of the iTRAQ ratios 115:114 < 0.67 and 117:115 < 0.67) after transfection with the miR-542-5p mimic. Supplementary Table 1 lists the proteins that are upregulated or downregulated in both the MNNG/HOS and U2OS cells. Figure 5C shows a heat map of the dysregulated proteins. MiRNAs can act as oncogenes or tumor suppressors by negatively regulating their downstream targets, and three downregulated proteins—HUWE1, CSE1L and PTBP1—were verified by western blot. The data showed that overexpression of miR-542-5p reduced levels of HUWE1, CSE1L and PTBP1, and inhibition increased levels of HUWE1, CSE1L and PTBP1 in both cell lines (Figure 5D, 5E). The relative expression of HUWE1, CSE1L and PTBP1 is shown in Supplementary Figure 4. Through the use of the bioinformatics tools, TargetScan, miRNAorg and miRwalk, HUWE1 was predicted to be the target gene of miR-542-5p. To determine whether miR-542-5p reduced HUWE1 expression through direct binding to its 3′UTR, we constructed the 3′UTR fragment of HUWE1, and the corresponding mutant counterpart was inserted directly downstream of the firefly luciferase reporter gene (Figure 5F). MiR-542-5p reduced the relative luciferase activity of the HUWE1-3′UTR binding site, whereas luciferase activity was not significantly changed in the mutant binding site (Figure 5G). These results suggested that miR-542-5p downregulated HUWE1 expression by directly targeting its 3′UTR.

### HUWE1 is the critical mediator of miR-542-5p in osteosarcoma cells

**Figure 6:**
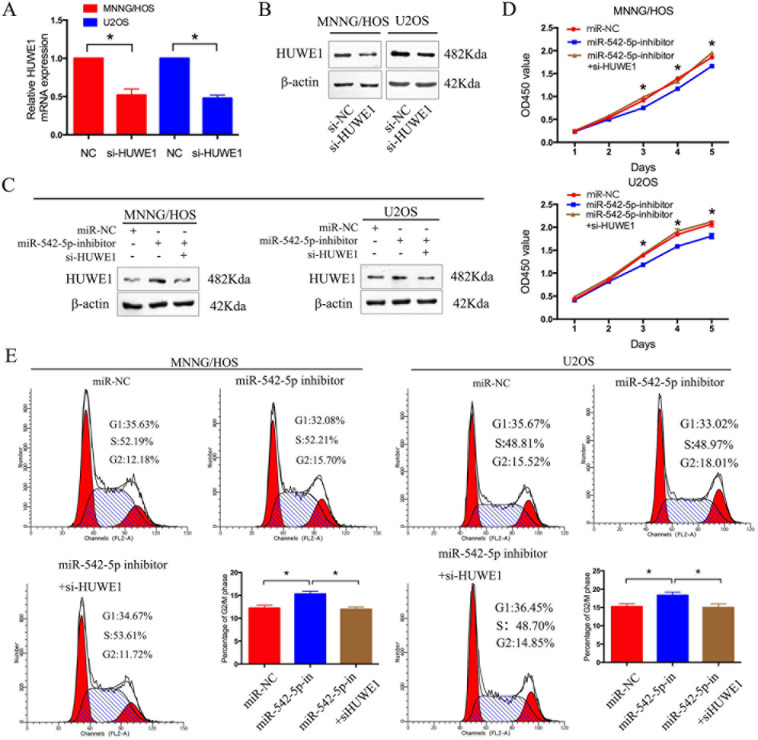
HUWE1 is the critical mediator of miR-542-5p in osteosarcoma cells **A. B.** Real-time PCR and western blot analyses of HUWE1 expression in MNNG/HOS and U2OS cells transfected with si-HUWE1 or the negative control. β-actin was used as an internal control. **C.** Western blot analysis of HUWE1 expression in MNNG/HOS and U2OS cells after transfection with anti-miR-NC, miR-542-5p inhibitors and si-HUWE1. **D.** CCK8 assays were determined after transduction with the miR-542-5p inhibitors, anti-miR-NC and si-HUWE1. **F.** Representative images and the table depict the results of cell cycle assays in MNNG/HOS and U2OS cells after transduction with the miR-542-5p inhibitors, anti-miR-NC and si-HUWE1. The data are representative of three independent experiments. Error bars represent SEM. * *P* < 0.05 by Student's *t* test, miR-NC VS miR-542-5p inhibitor; miR-542-5p inhibitor VS miR-542-5p inhibitor + si-HUWE1.

To verify the involvement of HUWE1 in the miR-542-5p-induced promotion of osteosarcoma cell proliferation, we knocked down endogenous HUWE1 expression in osteosarcoma cells using a specific siRNA. As shown in Figure 6A and 6B, si-HUWE1 significantly reduced levels of HUWE1 mRNA and protein. The relative expression of HUWE1 was shown in Supplementary Figure 5A. We then transfected si-HUWE1 and miR-542-5p inhibitors together into MNNG/HOS and U2OS cells. HUWE1 protein levels were analyzed by western blotting (Figure 6C). The relative expression of HUWE1 was shown in Supplementary Figure 5B. Knockdown of HUWE1 by siRNA in osteosarcoma cells transfected with miR-542-5p inhibitors attenuated the suppressive effects of miR-542-5p inhibitor on the proliferation of osteosarcoma cells (Figure 6D, 6E).

### MiR-542-5p is inversely correlated with HUWE1 in osteosarcoma tissues

**Figure 7:**
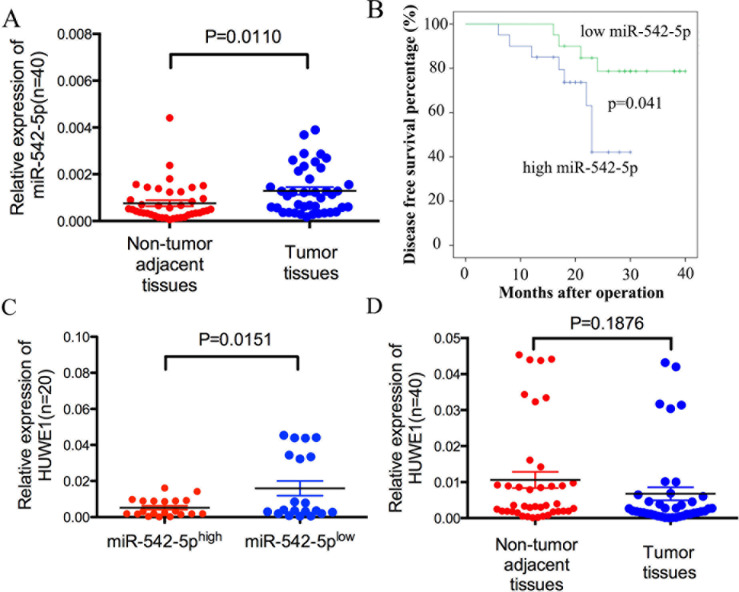
MiR-542-5p expression is inversely correlated with levels of HUWE1 in osteosarcoma **A.** Real-time PCR analysis to quantify the endogenous levels of miR-542-5p in osteosarcoma patients compared with paired noncancerous tissues. **B.** The prognostic value of miR-542-5p for osteosarcoma patients assessed by Kaplan-Meier analysis. **C.** The expression of HUWE1 was measured in the presence of low and high miR-542-5p expression levels. **D.** Real-time PCR analysis to quantify the endogenous levels of HUWE1 in osteosarcoma patients compared with paired noncancerous tissues.

We used quantitative real-time PCR (qRT-PCR) to measure the expression of miR-542-5p in 40 pairs of human tissue samples; each pair comprised an osteosarcoma sample and a corresponding non-tumor tissue sample. Compared with non-tumor tissue, miR-542-5p expression was upregulated in osteosarcoma (Figure 7A). A Kaplan-Meier analysis revealed a significant difference in disease free survival time between the high miR-542-5p group and low miR-542-5p group (χ2=4.193, P=0.041) (Figure 7B). Moreover, HUWE1 levels were negatively correlated with miR-542-5p expression in osteosarcoma tissues (Figure 7C). However, HUWE1 levels had no significant difference between the tumor tissues and non-tumor adjacent tissues (Figure 7D).

## DISCUSSION

Osteosarcoma is a type of malignant bone tumor characterized by high levels of genetic instability, with a peak incidence in the second and third decades of life [12]. Since the invention of pre- and postoperative chemotherapy in the 1970s, the 5-yearevent-free survival (EFS) rate of patients with localized disease has reached 70%. However, the prognosis for patients with metastatic osteosarcoma is poor, with 5-year EFS rates of no more than 20% [13]. MiRNAs reportedly contribute to the development and metastasis of osteosarcoma [14], and may provide novel therapeutic targets for treatment of this cancer.

In this study, microarray analysis was used to study the effects of TSA, which had previously been reported to inhibit osteosarcoma growth [10], on osteosarcoma miRNA expression profiles *in vitro*. Fifteen promising miRNAs were selected for validation by qRT-PCR analysis, several of which were previously reported to be involved in the tumorigenesis of osteosarcoma. MiR-195-5p and miR-374a-5p were previously shown to be upregulated in osteosarcoma patients [15]. In addition, serum miR-199a-5p levels are reportedly higher in osteosarcoma patients than in controls [16], and MiRNA-199a-3p, which regulates cell proliferation and migration *in vitro*, is downregulated in osteosarcoma tissues [17].

After functional screening, we found that miR-542-5p promoted proliferation in osteosarcoma cells. Notably, the function of miR-542-5p appears to vary in different tumors. In neuroblastoma, for example, it appears to act as a tumor suppressor [18]. It also inhibits growth of human lung cancer cells, perhaps by targeting EGFR mRNA [19] and is downregulated in thyroid tumors, suggesting miR-542-5p acts as a tumor suppressor in these cancers [20]. Conversely, miR-542-5p was upregulated in rectal cancer and basal cell carcinomas, indicating that miR-542-5p may act as an oncogene in those cancers [21, 22]. Upregulation of miR-542-5p was also associated with EMT in human carcinosarcomas [23].

Our experimental data revealed that miR-542-5p promoted the proliferation of osteosarcoma cells, but did not influence metastasis. Further study showed that overexpression of miR-542-5p promoted tumorigenesis of osteosarcoma cells *in vivo*. Moreover, miR-542-5p expression was upregulated in human osteosarcoma tissue compared with corresponding non-tumor tissues. Kaplan-Meier analysis revealed a significant difference in disease free survival time between the high and low miR-542-5p groups. These data indicate that miR-542-5p exhibits tumor-promoting activity in osteosarcoma and predicts poor patient prognosis.

By regulating the expression of associated genes, MiRNAs regulate a wide range of biological functions, including cell proliferation, migration, differentiation and apoptosis [24]. As reported, a single miRNA can regulate hundreds of mRNAs, and a single mRNA might be regulated by multiple miRNAs [25]. Konno *et al*. revealed that EZH2, MCL-1 and FOS were direct targets of miR-101 through gene microarray analysis [26], while exogenous overexpression of miR-29a reduces cell proliferation and invasiveness in non-small cell lung cancer by modulating the expression of several downstream proteins [27]. Comparing protein coding gene expression following miRNA treatment is a good method to identify the novel target of miRNA. To identify the underlying mechanism by which miR-542-5p functions, in the present study iTRAQ combined with NanoLC-MS/MS analysis was used to identify the proteins differentially expressed after transfecting cells with a miR-542-5p mimic. The results showed that seven proteins were downregulated and nine were upregulated in both MNNG/HOS and U2OS osteosarcoma cell lines. Among the downregulated proteins were HUWE1, CSE1L and PTBP1. It was previously reported that HUWE1 inhibits Wnt signaling [28] and suppresses Ras-mediated tumorigenesis by preventing accumulation of c-Myc/Miz1 complexes, which mediate p21 and p15 downregulation [29]. CSE1L reportedly facilitates cell death caused by a form of stress linked to the tumor suppressor p53 [30]. PTBP1 was shown to play a role in maintaining the growth and malignant properties of breast cancer cells [31]. Changes in protein levels were verified by western blot. Data from the present study showed that overexpression of miR-542-5p reduced levels of HUWE1, CSE1L and PTBP1, while inhibition of miR-542-5p increased levels of these proteins. Dual-luciferase reporter assays showed HUWE1 is a direct target of miR-542-5p in osteosarcoma. Moreover, levels of miR-542-5p and HUWE1 are inversely correlated in osteosarcoma tissues. However, there was no significant difference between HUWE1 levels in tumor tissues and non-tumor adjacent tissues.

In conclusion, the results obtained in the present study indicate that overexpression of miR-542-5p promotes the proliferation of osteosarcoma *in vitro* and *in vivo*. Further investigation revealed that HUWE1 was a direct target of miR-542-5p, and that reduced expression of this protein played an important role in the tumor-promoting effect of miR-542-5p overexpression. Importantly, these findings indicate that miR-542-5p may be a promising new therapeutic target for suppression of osteosarcoma proliferation.

## MATERIALS AND METHODS

### Cell lines and cell culture

Two osteosarcoma cell lines were used: MNNG/HOS and U2OS. The cells were maintained at 37°C in a humidified atmosphere containing 5% CO_2_. Cells were cultured in Dulbecco's modified Eagle's medium (DMEM) (MNNG/HOS cells) or RPMI-1640 (U2OS cells) and supplemented with 10% fetal bovine serum (FBS) (Biowest, South America Origin), 100 U/ml penicillin (Sigma-Aldrich, St Louis, MO, USA) and 100 mg/ml streptomycin (Sigma-Aldrich).

### Human osteosarcoma samples

Between 2011 and 2012, a total of 40 human osteosarcoma samples and the adjacent non-tumor tissue were collected during surgery at the Department of Orthopedics, Shanghai Jiao Tong University Affiliated Sixth People's Hospital (Shanghai, China). Upon resection, the human specimens were immediately frozen in liquid nitrogen and stored at −80°C. Informed consent was obtained from all patients, and the study was approved by the Ethics Committee of the Shanghai Jiao Tong University Affiliated Sixth People's Hospital.

### MiRNA microarray analysis

MiRNA expression profiling was carried out according to the manufacturer's instructions. Briefly, the cells were treated for 48 h with either 1% DMSO or 100 nmol/L TSA, and miRNAs were isolated using a mirVana miRNA Isolation Kit (Ambion, Carlsbad, CA, USA) according to the manufacturer's instructions. The quantity and quality of the RNA was determined by absorbance at 260 and 280 nm, and 100 ng of total RNA was used for microarray analysis. The RNA was labeled and hybridized onto the Agilent One-Color Microarray-Based miRNA Expression Analysis platform (Oe BioTech Co.). Following hybridization, the slides were washed, dried and scanned on an Agilent Scanner. The microarrays were independently performed three times. Clustering of the miRNA expression data was performed using CLUSTER. The differentially expressed miRNAs underwent GO (Gene Ontology) analysis and KEGG (Kyoto Encyclopedia of Genes and Genomes) pathway analysis. Three biological replicates were included.

### Oligonucleotide transfection

The miRNA mimics and control miR that were used for transient transfection were designed and synthesized by RiboBio (Guangzhou, China). The miRNA inhibitors and small interfering RNAs (siRNA) were synthesized by Biomics Biotechnologies (Nantong, China). A total of 5×10^4^ cells were seeded into the wells of a 6-well plate. The next day, the cells were transfected with the miRNA mimic or miRNA inhibitor using Lipofectamine 2000 Reagent (Invitrogen) according to the manufacturer's protocol. For assays of proliferation, cell cycle, migration and invasion, and for RNA extraction and western blotting, cells were used 48 h after transfection.

### Cell proliferation assays and cell cycle analysis

Cell proliferation: Forty-eight h after transfection, 5000 cells were seeded into each well of a 96-well plate and incubated. A 10-μL aliquot of CCK-8 (Dojindo, Japan) was added to quintuplicate wells and incubated for 2 h. To calculate the number of viable cells in each well, the absorbance at 450 nm was measured. Each measurement was performed in quintuplicate, and the experiments were repeated twice. Cell cycle: Forty-eight h after transfection, the cells were fixed in 70% ethanol at −20°C for 24 h. Then, the cells were stained with 50 μg/mL propidium iodide (PI) (Kaiji, China) and analyzed using a FACSCalibur flow cytometer (BD Biosciences, San Jose, CA). The results were analyzed using ModFit software (BD Biosciences, San Jose, CA). Assays were independently conducted three times.

### *In vitro* migration and invasion assays

Cell migration and invasion assays were performed in a 24-well plate with 8-mm pore size chamber inserts (Corning, New York, NY, USA). For the migration assays, after transfection with either the mimic or control miR, 5×10^4^ cells per well were placed into the upper chamber on an uncoated membrane. For the invasion assays, after transfection with either the mimic or control miR, 1×10^5^ cells per well were placed into the upper chamber on a Matrigel-coated membrane. The cells were diluted with serum-free culture medium. In both assays, when the cells were seeded into the upper chamber, they were suspended in 200 μl of DMEM without FBS. The lower chambers contained 800 μL of medium with 10% FBS. The cells were incubated at 37°C in 5% CO_2_ for 12 h and 14 h for the migration and invasion assays, respectively. Then, the membrane inserts were removed and non-invading cells were removed from the upper surface of the membrane. Cells that had moved to the bottom of the chamber were fixed with 100% methanol for 30 min and stained with 0.1% crystal violet for 30 min. The cells in at least 10 random fields were imaged and counted using a CKX41 inverted microscope (Olympus, Tokyo, Japan). Assays were independently conducted three times.

### RNA isolation and quantitative real-time PCR assays

MiRNA was extracted from cultured cells and human tissue samples using a mirVana miRNA Isolation Kit (Ambion, Carlsbad, CA, USA). The expression levels of the mature miRNAs were quantified with specific primers and probes using TaqMan miRNA assays (Applied Biosystems, Carlsbad, CA, USA) according to the manufacturer's instructions, and were normalized to the U6 small nuclear RNA. Total RNA was extracted from transfected cells and human tissues using TRIzol reagent (Invitrogen, Carlsbad, CA, USA) according to the manufacturer's protocol, and was quantified with a NanoDrop 2000 (Thermo Fisher Scientific, Waltham, MA, USA). First-strand cDNA was synthesized with a PrimeScript RT Reagent Kit (TaKaRa, Shiga, Japan). RT-PCR was performed with SYBR Green Premix Ex Taq (TaKaRa). The primer sequences were as follows: HUWE1 (forward primer: 5′-ACTGGTGCAACTTCCTCCTT-3′; reverse primer: 5′-TTGTCCTGGGCTGCAATCTC-3′). β-actin (forward primer: 5′-GTCATTCCAAATATGAGATGCGT-3′; reverse primer: 5′-GCATTACATAATTTACACGAAAGCA-3′) was used as an internal control.

### Stable expression of miR-542-5p using lentiviral vectors

The miR-542-5p-mimic, miR-542-5p-inhibitor and miR-control lentiviral vectors were purchased from GenePharma (Shanghai, China). The sequences are as follows: miR-542-5p-mimic lentiviral vector (5′-TCGGGGATCATCATGTCACGAGA-3′); miR-542-5p-inhibitor lentiviral vector (5′-TCTCGTGACATGATGATCCCCGA-3′); miR-control lentiviral vector (5′-TTCTCCGAACGTGTCACGT-3′). MNNG/HOS cells were transfected with the miRNA-mimic lentiviral vector, the miRNA-inhibitor lentiviral vector or the miRNA-NC lentiviral vector in the presence of 5 mg/ml Polybrene (GenePharma).

### Colony formation assay

1×10^3^ MNNG/HOS cells stably expressing the miR-542-5p mimic, miR-542-5p inhibitor or miR-control were seeded in 6-well plates. After two weeks, cells in each well were fixed with 100% methanol for 30 min and stained with 0.1% crystal violet for 30 min. Cell colonies were then counted. Assays were independently conducted three times.

### Animal experiments

All animal experiments were approved by the Ethics Committee of the Shanghai Jiao Tong University Affiliated Sixth People's Hospital. For tumor growth assays, MNNG/HOS cells stably expressing the miR-542-5p mimic, miR-542-5p inhibitor or miR-control were injected subcutaneously into the left scapulas of nude mice (6-week-old BALB/c-nu/nu, 6 per group, 2×10^6^ cells per mouse). The tumor volume was monitored weekly and was calculated using the formula: V =0.5×length×width^2^. After 6 weeks, the mice were killed.

### Proteomic analysis

iTRAQ combined with NanoLC−MS/MS analysis [11] was used to identify proteins that were differentially expressed after transfection with the miR-542-5p mimic. MNNG/HOS and U2OS cells were transfected with the miR-542-5p mimic or with miR-NC for 48 h and harvested, and 100 μg of the MNNG/HOS cell lysates were labeled with iTRAQ labeling reagents 115 (miR-542-5p mimic) and 114 (miR-NC) (Applied Biosystems, Foster City, CA). Similarly, whole-cell lysates from U2OS cells transfected with the miR-542-5p mimic or miR-NC were labeled with iTRAQ labeling reagents 117 and 116 (Applied Biosystems, Foster City, CA), respectively. After strong cation exchange and NanoLC−MS/MS analysis, protein identification and iTRAQ quantitation were performed using ProteinPilot4.1 software (AB SCIEX, USA). Two physical replicates were performed. Figure 5A shows the flow chart of proteomic analysis.

### Western blot analysis

Lysates were extracted from cultured cells using a mixture of T-PER Protein Extraction Reagent (Thermo Fisher Scientific), PhosSTOP (Roche, Basel, Switzerland) and Complete Mini (Roche). The protein samples were separated using 6% and 8% sodium dodecyl sulfate-polyacrylamide gel electrophoresis and were transferred to nitrocellulose membranes (Millipore, Billerica, USA). After blocking in phosphate-buffered saline/Tween-20 containing 5% non-fat milk, the membranes were incubated with the following primary antibodies: HUWE1 (Bioworld Technology, 1:500), CSE1L (Bioworld Technology, USA, 1:500), PTBP1 (Bioworld Technology, 1:500) or β-actin (Sigma-Aldrich, 1:20000). The secondary antibody was anti-rabbit IgG (Sigma-Aldrich, 1:5000). Subsequent visualization was performed with SuperSignal West Femto Maximum Sensitivity Substrate (Thermo Fisher Scientific).

### Dual-luciferase reporter assay

A mixture of 50 ng pluc-3′UTR, 10 ng Renilla and 5 pmol miRNA-542-5p mimic or negative control were co-transfected into HEK-293T cells according to the recommended instructions using Lipofectamine 2000. After 48 h of transfection, Firefly and Renilla luciferase activity was measured by the Dual-Luciferase Reporter Assay System (Promega). The relative firefly luciferase activities were detected by normalizing to Renilla luciferase activities, which served as an internal control for transfection efficiency.

### Statistical analysis

Data were imaged with GraphPad Prism 5 software (GraphPad Software, Inc., La Jolla, CA, USA). Quantitative variables are presented as means and standard error of the mean and were analyzed using Student's t-tests (two-tailed; P < 0.05 was considered statistically significant). The Kaplan-Meier analysis was employed for the survival analysis between the groups. The disease free survival time is the period from surgery to the presence of new lesions. All analyses were performed using SPSS software (version 19.0) (IBM Corporation, New York, NY, USA).

## SUPPLEMENTARY MATERIAL TABLE AND FIGURES


