# Supplementary material for: MiR-542-5p is a negative prognostic factor and promotes osteosarcoma tumorigenesis by targeting HUWE1
Source: Oncotarget. 2015 Oct 20;6(40):42761–72. doi: 10.18632/oncotarget.6199 (PMC4767468; doi:10.18632/oncotarget.6199)
Supplement: Supplementary file 1 [file oncotarget-06-42761-s001.pdf]

MiR-542-5p is a negative prognostic factor and promotes osteosarcoma tumorigenesis by targeting HUWE1

Supplementary Material

Supplementary table 1. The list of the proteins that were upregulated or downregulated in both the MNNG/HOS and U2OS cells.

Accession number	Protein description	Gene name	115:114 (mean±SD)	117:116 (mean±SD)
sp|P26599|PTBP1	Polypyrimidine tract-binding protein 1	*PTBP1*	0.32±0.14	0.57±0.04
sp|P35527|K1C9	Keratin, type I cytoskeletal 9	*KRT9*	0.07±0.00	0.19±0.00
sp|P55060|XPO2	Exportin-2	*CSE1L*	0.30±0.01	0.40±0.09
sp|P63010|AP2B1	AP-2 complex subunit beta	*AP2B1*	0.46±0.13	0.64±0.03
tr|C9J9K3|C9J9K3	40S ribosomal protein SA (Fragment)	*RPSA*	0.42±0.06	0.53±0.05
tr|E9PR17|E9PR17	CD59 glycoprotein	*CD59*	0.53±0.12	0.39±0.33
tr|H0Y7U1|H0Y7U1	E3 ubiquitin-protein ligase HUWE1 (Fragment)	*HUWE1*	0.44±0.00	0.50±0.00
sp|O00273|DFFA	DNA fragmentation factor subunit alpha	*DFFA*	1.81±0.42	6.08±5.94
sp|P02768|ALBU	Serum albumin	*ALB*	2.32±0.58	2.16±0.36
sp|P36543|VATE1	V-type proton ATPase subunit E 1	*ATP6V1E1*	2.24±0.69	1.57±0.08
sp|Q96NY7|CLIC6	Chloride intracellular channel protein 6	*CLIC6*	2.69±0.63	3.34±1.11
sp|Q96S82|UBL7	Ubiquitin-like protein 7	*UBL7*	2.74±0.94	3.02±0.78
sp|Q9NXG6|P4HTM	Transmembrane prolyl 4-hydroxylase	*P4HTM*	1.80±0.00	1.68±0.01
sp|Q9NZZ3|CHMP5	Charged multivesicular body protein 5	*CHMP5*	3.17±0.94	2.05±0.25
sp|Q9Y4D1|DAAM1	Disheveled-associated activator of morphogenesis 1	*DAAM1*	4.21±0.00	2.68±0.00
tr|Q75MJ1|Q75MJ1	ATP-binding cassette sub-family F member 2	*ABCF2*	1.73±0.17	1.95±0.06

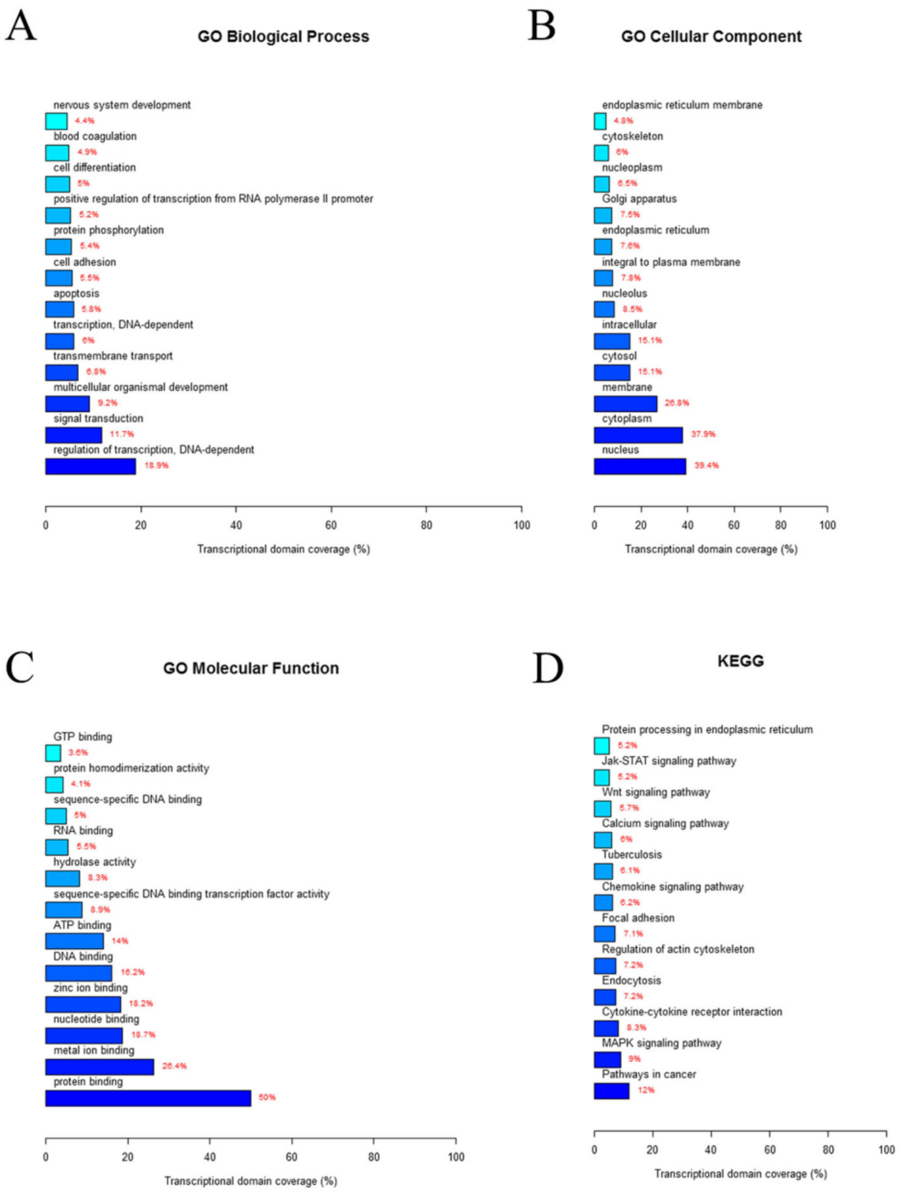

Supplementary Figure 1. GO analysis and KEGG analysis of differentially expressed miRNAs.

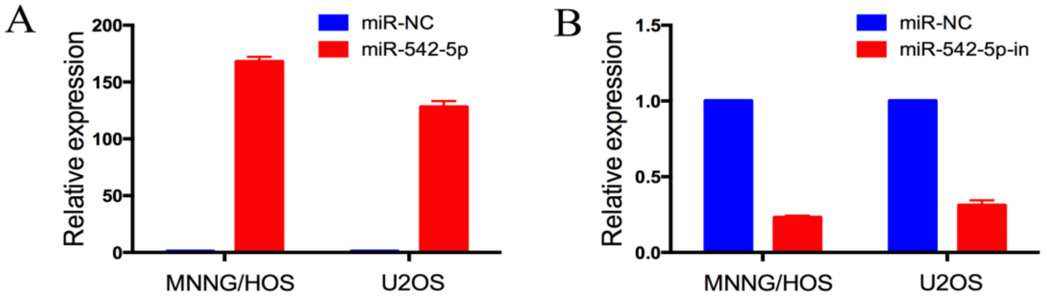

Supplementary Figure 2. The relative expression of miR-542-5p after transfection with mimics or inhibitors in MNNG/HOS and U2OS cells.

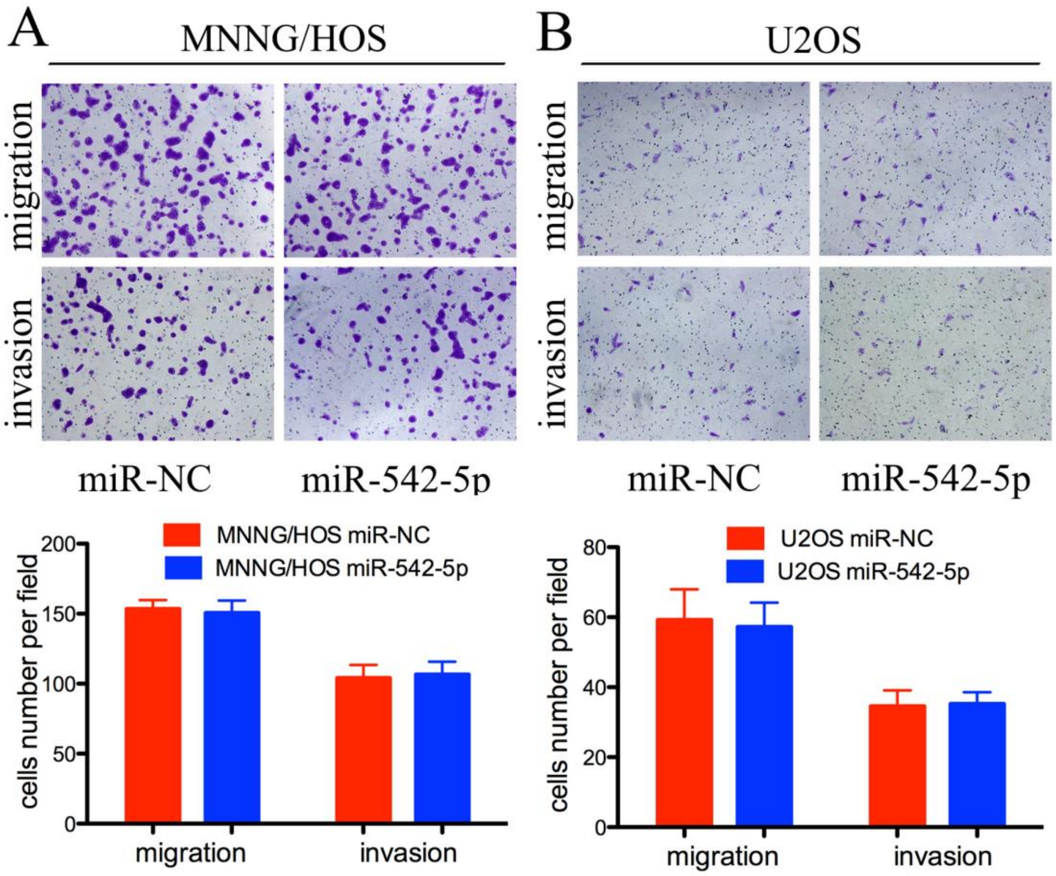

Supplementary Figure 3. Transwell assays were used to detect the migration and invasion capability of each cell line after transfection with a miR-542-5p mimic. The upper panels show representative photos (magnification: 100×) of invasive cells, and the lower panel shows histograms of the results. Statistical analysis was performed using Student’s t-test (n = 3).

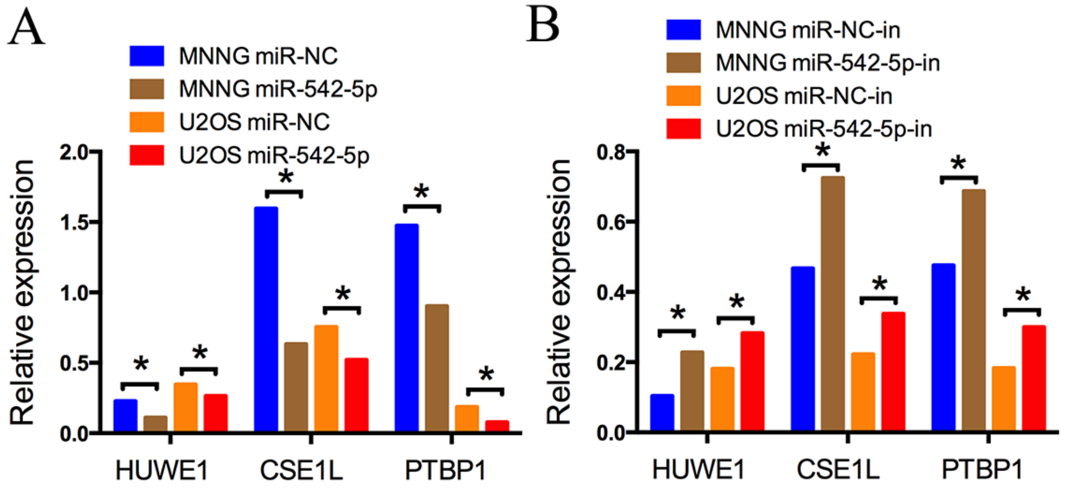

Supplementary Figure 4. The relative expression of HUWE1, CSE1L and PTBP1 after transfection with miR-542-5p mimics or inhibitors in MNNG/HOS and U2OS cells.

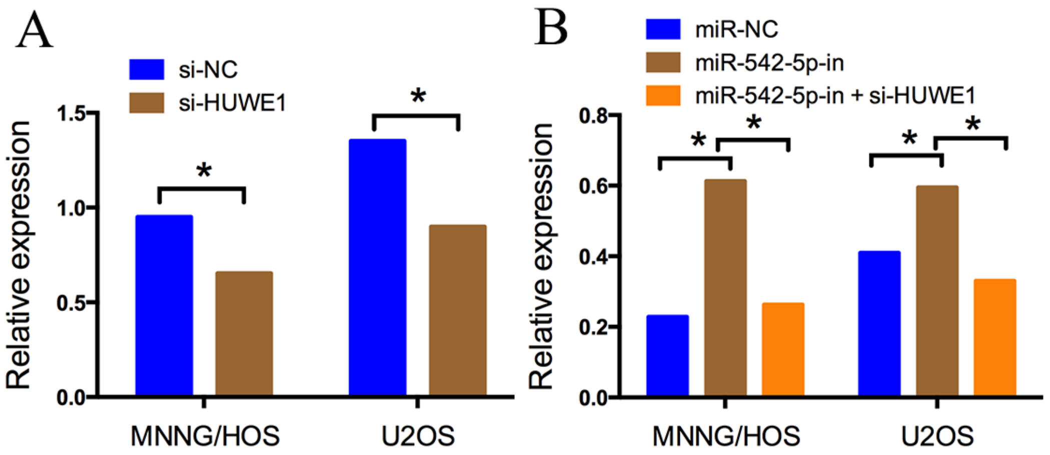

Supplementary Figure 5. (A) The relative expression of HUWE1 after transfection with siRNA in MNNG/HOS and U2OS cells. (B) The relative expression of HUWE1 in MNNG/HOS and U2OS cells after transfection with anti-miR-NC, miR-542-5p inhibitors and si-HUWE1.
